# Diphtheria in Pakistan post-COVID-19, a potential public health threat: an update

**DOI:** 10.1186/s41182-023-00522-y

**Published:** 2023-05-11

**Authors:** Mahnoor Saeed, Muhammad Bilal Shahid, Aroma Naeem, Shehroze Tabassum, Tirth Dave

**Affiliations:** 1 King Edward Medical University, Lahore, Pakistan; 2 Bukovinian State Medical University, Chernivtsi, Ukraine

## Abstract

Diphtheria, a vaccine-preventable disease, remains a concern in Pakistan as cases have risen post-COVID-19 pandemic causing more than 45 deaths in Pakistan in the year 2022. The respiratory variant of the disease is more common and can lead to serious complications, such as myocarditis and respiratory insufficiency. Diphtheria has caused havoc in the past killing millions of people worldwide before the development of its vaccine. Although the diphtheria toxoid vaccine is effective against toxigenic strains, there have been cases of treatment-resistant strains, particularly the non-toxigenic strains of C. diphtheriae. Pakistan's economic and health systems have suffered setbacks, which have been exacerbated by the COVID-19 pandemic. The pandemic has disrupted routine vaccination programs, and recent floods have contributed to an increase in diphtheria cases and rendered millions homeless. Poor immunization services, inadequate training of vaccination teams, and wealth inequality have all contributed to unequal vaccination coverage in Pakistan. The rising cases of diphtheria call for prompt action, including booster shots, updating vaccination records and administering immediate doses of the toxoid to close contacts.

## The rising incidence of diphtheria yet again in Pakistan post-COVID-19 pandemic: a call for concern

Dear Respected Editor,

Diphtheria is a vaccine-preventable but potentially fatal infection, having respiratory and non-respiratory variants of which the former is more prevalent and commonly presents with fever, malaise, sore throat and swollen lymph nodes. Greyish-white pseudomembrane formation on the mucosa of the nose and throat is the hallmark of respiratory diphtheria (RDP) [1]. The 5–10% mortality rate of DP, despite treatment [1], the airborne mode of transmission makes it a disease we cannot turn a blind eye to. DP wreaked havoc in the pre-vaccine era causing millions of deaths worldwide until the development of the toxoid vaccine in 1923 [2]. Strenuous efforts made at the global level in the following years resulted in a significant decline in the total number of cases; however, the disease could not be eradicated. The epidemic resurfaced time and again in European countries [3] and Russia in the 1990s [4]. According to the World Health Organization (WHO), the number of deaths brought about by diphtheria approximated to 10,000 people during 2000–2004 and the number inclined to 5000 people during 2005–2015 worldwide [2].

The causative factor is the toxigenic strains of bacteria of the Corynebacterium genus, mostly commonly *C. diphtheria*. However, in recent years, non-toxigenic strains of C. diphtheriae (NTCD) have also been recognized as potentially emerging pathogens causing respiratory and systemic manifestations of DP [5]. A few complications of DP include myocarditis, neuritis, otitis media and respiratory insufficiency due to paralysis of the diaphragm [1]. Diagnosis of DP is primarily clinical; however, a throat swab can be used to culture the bacteria for diagnostic confirmation. Antimicrobial agents such as penicillins and macrolides, and anti-diphtheria serum are effective therapies for combating acute infection; however, treatment-resistant cases, particularly of NTCD strains, have also been reported [6]. The Diphtheria toxoid vaccine has proven to be immensely effective against these toxigenic strains. In Pakistan, diphtheria toxoid became a part of routine vaccination in children at 6, 10, and 14 weeks of age, with the introduction of the Extended Program of Immunization in 1978.

**Fig. 1:**
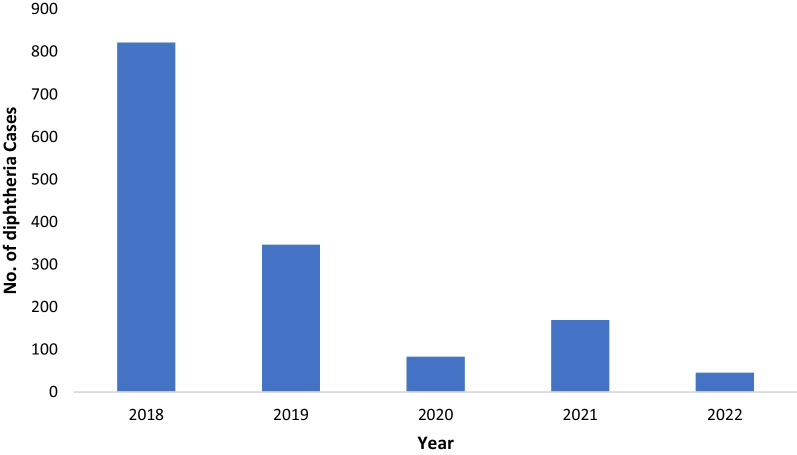
Showing diphtheria cases in Pakistan during last 5 years

South East Asia has been the leading region of worldwide DP incidence during the last decade [2]. After India, Pakistan reported the highest number of cases from the South Asia region in 2021 [7]. During the last year, 2022, we have again seen a surge of DP cases in Pakistan, which has the potential to leave the country in shambles. In 2022, the Ministry of Health Services, Regulations and Coordination (NHS, R&C) reported the death of more than 45 children due to DP in Pakistan [8]. The number of diphtheria cases in Pakistan during the last 5 years is illustrated in Fig. 1 [7, 8].

Being a developing country, Pakistan has faced a devastating economic downfall over time which has badly affected its already fragile health system. The COVID-19 pandemic, followed by lockdown periods, and travel restrictions disturbed the routine vaccination program of the country [9]. People were reluctant to visit doctors for routine checkups and vaccination of their children due to the fear of catching the COVID-19 virus [10]. Recent floods in 2022 have made millions of people homeless resulting in a breakthrough in the number of cases of DP, mumps, and other similar illnesses in an already exposed environment [10]. The continuous seasonal migration of people living below the poverty line puts them at a disadvantage in keeping track of the vaccination statuses of the children living in a specific area. In the past, flaws in vaccine quality, availability, and transportation in Pakistan have also contributed to the hindrance in the treatment of diphtheria [11]. Furthermore, immunization services in Pakistan have been unsatisfactory due to a lack of accountability, political interference, inadequate training of vaccination teams, and poor maintenance of the cold chain [12]. Unequal vaccination coverage by the programs of underdeveloped countries like Pakistan due to wealth inequality in the masses is also a distressing concern [13]. A study conducted by Xavier Bosch-Capblanch revealed that according to the Data Quality Audit (DQA), the immunization data of Pakistan was insufficient and unreliable [14]. Shortage of Diphtheria Antitoxin was noticed recently by WHO when, despite the increase in demand, the National Institute of Health (NIH), Islamabad could not manufacture the anti-diphtheria serum (ADS) owing to the lack of modern equipment [15].

The rising cases of diphtheria pose a serious threat to Pakistan's public health and thus warrant prompt courses of action. The immunity conferred by diphtheria toxoid declines over time, hence, implementation of booster shots every 10-year interval and their up-to-date entry in the national database can improve herd immunity. The vaccination history of close contacts is of prime importance. If the vaccination history of any contact is unknown, incomplete or the last dose was administered more than 5 years ago, then the contact must receive an immediate dose of the toxoid. A 7–10-day course of oral macrolide or a single intramuscular dose of penicillin G is advised for close contact [1]. The use of modern technology like mobile phones for reminding the masses about upcoming routine immunization (RI) visits has shown positive results in the past [16]. Revival of such programs can increase vaccination coverage. Vaccination records of diphtheria and similar illnesses should be digitalized against computerized national identity card (CNIC) numbers and a five-digit biometric verification system should be introduced to efficiently monitor vaccine administration, especially among the children of the nomads and lower class who have more chances of losing their vaccination cards. Gaps in vaccination due to COVID-19 and floods should be filled by educating the population regarding catch-up vaccination [17] and expanding the outreach services to locate unvaccinated children. To curb this menace at its root, we need to strengthen our primary health care system in rural areas to fight the misconceptions of the general population regarding vaccination in underdeveloped regions with evidence and ensure adequate immunization services in far-off regions. Government policies should be modified to increase the healthcare budget and facilitate research programs and surveillance systems to gather reliable data for better monitoring and policy-making in the future. WHO and United Nations Children's Fund (UNICEF) should take notice of the diphtheria surge in especially low-income countries like Pakistan and collaborate with the government of Pakistan to ensure the enforcement of effective measures. Such efforts can help us to alleviate the burden of DP in Pakistan.

## Data Availability

Not applicable.
